# The Out-of-pocket Expenses of People With Tinnitus in Europe

**DOI:** 10.2188/jea.JE20230358

**Published:** 2024-11-05

**Authors:** Carlotta M. Jarach, Kyriaki Karydou, Ilias Trochidis, Alberto Bernal-Robledano, Piet A. van den Brandt, Rilana Cima, Christopher R. Cederroth, Jose Antonio Lopez-Escamez, Simone Ghislandi, Deborah A. Hall, Dimitris Kikidis, Berthold Langguth, Alessandra Lugo, Birgit Mazurek, Anna Odone, Martin Schecklmann, Stefan Schoisswohl, Jorge P. Simoes, Winfried Schlee, Silvano Gallus

**Affiliations:** 1 Istituto di Ricerche Farmacologiche Mario Negri IRCCS, Department of Medical Epidemiology, Milan, Italy; 2 ViLabs, Limassol, Cyprus; 3 Instituto de Investigación Biosanitaria ibs.GRANADA, Granada, Spain; 4 Maastricht University Medical Centre, CAPHRI- School for Public Health and Primary Care, Department of Epidemiology, Maastricht, the Netherlands; 5 Maastricht University Medical Centre, GROW- School for Oncology and Developmental Biology, Department of Epidemiology, Maastricht, the Netherlands; 6 Health Psychology, Faculty of Psychology and Educational Sciences, KU Leuven University, Leuven, Belgium; 7 Tinnitus Center of Expertise, Centre of Expertise in Rehabilitation and Audiology, Adelante, Hoensbroek, The Netherlands; 8 Experimental Health Psychology, Faculty of Psychology and Neurosciences, Maastricht University, Maastricht, The Netherlands; 9 Laboratory of Experimental Audiology, Department of Physiology and Pharmacology, Karolinska Institutet, Stockholm, Sweden; 10 Translational Hearing Research, Tübingen Hearing Research Center, Department of Otolaryngology, Head and Neck Surgery, University of Tübingen, Tübingen, Germany; 11 Sensorineural Pathology Programme, Centro de Investigación Biomédica en Red en Enfermedades Raras, CIBERER, Madrid, Spain; 12 Meniere’s Disease Neuroscience Research Program, Faculty of Medicine & Health, School of Medical Sciences, The Kolling Institute, University of Sydney, Sydney, New South Wales, Australia; 13 Department of Social and Political Sciences, Bocconi University, Milano, Italy; 14 School of Social Sciences, Heriot-Watt University, Edinburgh, United Kingdom; 15 Department of Otorhinolaryngology, Head and Neck Surgery, National and Kapodistrian University of Athens, Medical School, Athens, Greece; 16 Department of Psychiatry and Psychotherapy, University of Regensburg, Regensburg, Germany; 17 Interdisciplinary Tinnitus Clinic, University of Regensburg, Regensburg, Germany; 18 Tinnitus Center, Charité Universitätsmedizin Berlin, Berlin, Germany; 19 School of Medicine, University Vita-Salute San Raffaele, Milan, Italy; 20 Department of Public Health, Experimental and Forensic Medicine, University of Pavia, Pavia, Italy; 21 Department of Psychology, Universität der Bundeswehr München, Neubiberg, Germany; 22 Department of Psychology, Health and Technology, University of Twente, Enschede, The Netherlands; 23 Institute for Information and Process Management, Eastern Switzerland University of Applied Sciences, St. Gallen, Switzerland

**Keywords:** tinnitus, out of pocket costs, economic burden, patients’ perspective, epidemiology

## Abstract

**Background:**

Despite the high frequency of tinnitus and its impact on wellbeing, little is known about its economic burden, and, to our knowledge, no data are available on out-of-pocket (OOP) expenses.

**Methods:**

In 2022, a survey was conducted on OOP costs of tinnitus. We enrolled 679 participants with slight, moderate, and severe tinnitus in Italy, United Kingdom, Netherlands, Germany, and Spain. We estimated annual OOP expenses for tinnitus-related healthcare visits, treatments, medications, and alternative medicine practices. Prevalence of tinnitus in the general population, obtained from a representative survey we conducted in Europe in 2017–2018, was used to generalize costs for people with any tinnitus at the national level.

**Results:**

OOP expenses were 368€ (95% confidence intervals [CI], 78€–690€), 728€ (95% CI, 316€–1,288€), and 1,492€ (95% CI, 760€–2,688€) for slight, moderate, and severe tinnitus, respectively, with annual expenditure of 565€ for people with any tinnitus: 209€ for healthcare visits; 93€ for treatments; 16€ for drugs; 64€ for hearing supporting systems; and 183€ for acupuncture, homeopathy, and osteopathy. Individuals with slight, moderate, and severe tinnitus expressed a willingness to invest 1.6, 4.3, and 7.0 times their monthly income, respectively, to achieve complete relief from tinnitus.

**Conclusion:**

This study offers for the first time insights into the OOP expenses incurred by individuals with tinnitus. OOP expenses exhibited substantial variations based on severity status, accounting for more than 17 billion € in the countries considered. In terms of financial burden, these findings align tinnitus to the recognized leading disabilities, including back pain and migraine.

## INTRODUCTION

Tinnitus, the conscious perception of a sound without an identifiable external source,^1^ is a prevalent phenomenon estimated to affect approximately 15% of the adult population globally, severely impacting 2.3%.^2^ In a non-negligible percentage, this symptom results in the so-called tinnitus disorder, when associated with emotional distress, cognitive dysfunction, functional disability, and reduced quality of life.^1^

To date, there is no definitive cure; however, multiple treatment options have been proposed, with some of them showing efficacy in certain patients.^3^ The great number of potential therapies and the considerable quantity of comorbidities make tinnitus a substantial financial burden. In fact, people with tinnitus commonly embark on long and complicated treatment journeys consisting of multiple cycles of caregiver referral and treatment.^4^^,^^5^ This process is not only strenuous but can potentially result in high expenses for patients, healthcare systems, and society.^6^

To our knowledge, there is only one systematic review which focused on the economic burden of tinnitus;^6^ this review identified a total of five studies, mostly conducted more than 10 years ago in the United States,^7^^,^^8^ the Netherlands,^4^^,^^9^ and the United Kingdom,^10^ providing data on tinnitus economic costs. Of the five included articles, four provided mean annual cost estimates per patient of 564–3,429€ for healthcare costs, 69–115€ for patient and family costs and 2,565–3,702€ for indirect costs, including productivity loss. After that review, only one further study attempted to estimate tinnitus-related socioeconomic costs in Germany,^11^ estimating that the mean socioeconomic cost per tinnitus patient in the country was 4,799€ per year, including 2,206€ covered by public healthcare (either carried by the patient privately or in the form of economic loss for indirect costs or productivity loss).

The intensive efforts to find some relief suggest that tinnitus may have a significant financial impact not only on the healthcare systems, but also on out-of-pocket (OOP) expenses of tinnitus sufferers. Given tinnitus high prevalence and heterogeneity of care approaches, as well as reimbursement schemes, it is important to estimate tinnitus associated costs by type of costs (ie, health system costs, OOP costs, and indirect costs). Therefore, we conducted a comprehensive investigation aimed at assessing in detail the expenses associated with tinnitus for slight, moderate, and severe tinnitus sufferers. Taking advantage of a representative survey conducted in 12 European countries, we were able to generalize annual expenses at national level.^12^

## METHODS

The main task of the Unification of Treatments and Interventions for Tinnitus Patients (UNITI) project was the conduction of a large randomized controlled trial (RCT) aimed at comparing established standard treatments performed alone or in combination.^13^^,^^14^ Within UNITI, we conducted a cross-sectional study aimed at evaluating the tinnitus OOP expenses in Europe. 110 participants comprised patients from UNITI-RCT^13^ and a further 569 individuals were recruited via tinnitus experts or patient associations. The survey was available online under the EC platform “EU survey” from the 1st of February, 2022 until the 12th of October, 2022. The study was approved by the local ethics committees of the clinical sites participating in the UNITI-RCT.^15^ For Italy, this study was approved by the ethics committee of Mario Negri Institute (ethics committee of Fondazione IRCCS Istituto Neurologico Carlo Besta, Milan, Italy) who notified that no preventative evaluation was required for the present study since anonymous data were collected (File number 37/2017). Informed consent was obtained from all participants. All records were stored safely and anonymously following the General Data Protection Regulation (GDPR) rules.

The survey tool was based on a questionnaire used in a previously conducted cost-study in the Netherlands^4^ and finalized by an interdisciplinary group of five tinnitus experts. In addition, three tinnitus patients were recruited and involved in refining the questions. Comments were analyzed to identify common issues and challenges to questions and their respective wording. The comments received suggested the need to reword few questions ensuring clarity. To be eligible for the study, the inclusion criteria required being aged ≥18 years and having experienced tinnitus in the last year.

The questionnaire was drafted in English and translated into five languages: Dutch, German, Greek, Italian, and Spanish. The final version of the questionnaire (eMaterial 1) included a total of 48 questions. The questionnaire had a recall period of 12 months. Tinnitus was assessed using a set of validated tinnitus-related questions,^16^ able to classify participants in having slight, moderate, or severe tinnitus, and used by authors in a multi-country representative survey: the European Tinnitus Survey (ETS).^12^ In addition, data were gathered for the direct medical and non-medical OOP costs of participants’ due to tinnitus that were not covered by the national healthcare system or by private insurance. OOP costs were collected in local currency and converted in € (1 € equals 0.979 US dollars).

For calculating OOP expenses (average individual cost contribution per appointment in local currency) participants were asked to indicate the number of visits to healthcare professionals (we included preselected healthcare professionals as well as an open question to add possible other healthcare professionals) over the past 12 months as well as the average OOP cost per appointment. Participants were asked about the annual frequency and corresponding OOP costs for tinnitus treatments, such as drugs, hearing aids or support systems, and selected other activities that might have followed (ie, acupuncture, homeopathy, osteopathy).

To measure quality of life, participants were asked to indicate, on a scale from 0 to 100, where 100 marked the best state of health imagined and 0 the worst state, how good or bad they state their current health. Participants had to indicate if they had any paid job within the past 12 months; those with a paid job had to indicate the frequency and the number of days/months of reporting sick during this period due to tinnitus. We measured willingness to pay (WTP) to reduce their tinnitus loudness/annoyance either completely or by 50%. Corresponding responses ranged from “a quarter of my monthly income” to “over 20 times my monthly income”.

To generalize OOP expenses related to tinnitus found in our study at national level, we used data from the ETS, which is a representative cross-sectional study conducted in 2017–2018 in 12 selected European Union (EU) countries (Bulgaria, England, France, Germany, Greece, Ireland, Italy, Latvia, Poland, Portugal, Romania, and Spain) on a sample of 11,427 adults aged ≥18 years.^12^ The ETS, coordinated by Mario Negri Institute, University of Nottingham, and University of Regensburg, was conducted using a standardized set of tinnitus-related questions in country-specific languages.^16^ The same questions were also used in this survey on tinnitus OOP expenses. Participants without tinnitus were those answering “no” to the question: “Over the past year, have you had noises (such as ringing or buzzing) in your head or in one or both ears that lasts for more than 5 minutes at a time?”. Those with tinnitus (ie, answering “yes” to the previous question) were further investigated about tinnitus severity with the question: “Over the past year, how much do these noises in your head or ears worry, annoy or upset you when they are at their worst?” and were categorized as “slight”, “moderate”, or “severe”. Prevalence estimates for Germany, Italy, and Spain were directly derived by the country-specific ETS data; estimates for the United Kingdom were derived by ETS data for England; estimates for the Netherlands were estimated by those of the 12 EU countries combined (Table 1).

**Table 1.** Distribution of subjects aged 18 years or more from 5 European countries according to the presence and severity of tinnitus. European Tinnitus Survey (ETS), 2017–2018

	*N*	Tinnitus absent	Slight	Moderate	Severe
		%	%	%	%
Netherlands^*^	11,427	85.4	8.7	4.7	1.3
Germany	912	88.1	6.5	4.5	1.0
Italy	979	86.4	8.4	4.5	0.8
Spain	1,000	84.5	10.7	3.1	1.7
United Kingdom	1,007	84.7	9.8	4.6	0.9

^*^The ETS was not conducted in the Netherlands. For this country, we used the overall estimate of the 12 countries within the ETS.

We performed a comprehensive assessment of healthcare annual utilization, encompassing (1) healthcare visits, (2) treatments, (3) medication usage, (4) adoption of hearing aids or supporting systems, and (5) engagement in selected activities to reduce tinnitus. In this study, we utilized the generic term ‘item’ to refer to elements in the categories from 1 to 5. To ascertain the OOP mean expenses for each of these items, the following steps were performed: (i) proportion calculation: we determined the proportion of individuals who had engaged in each item stratified by severity status; (ii) mean cost calculation: the mean cost for each item was computed as the mean cost indicated as private expenses per specific item among those using such item in the overall sample (for the United Kingdom, an exchange rate of 1.172 was applied); (iii) average number calculation: when feasible, the average number of items was computed, again stratified by severity status; (iv) subsequently, the “OOP mean expenses per person” for each item was derived by multiplying the results obtained in steps (i), (ii), and (iii) for that specific item. We used the boot function in R to perform bootstrap resampling and then calculate the percentile 95% confidence intervals (CI) on the bootstrap means.

For the items in the category “hearing aids or supporting systems”, we did not specify a recall period of 12 months, limiting the questions to the mere use and their total cost. To standardize this expense over a 1-year period, we assumed a 4-year lifespan for the devices and applied a 25% annual depreciation rate. Thus, the OOP mean expenses per person (iv) were calculated by multiplying the proportion (i), the OOP mean cost (ii), with this 25% annual depreciation rate. This entire process was subsequently replicated for females and males and each single country separately.

To assess the average number of sick leave days taken across severity levels of tinnitus, we applied the average sick leave duration observed in the sample to all categories.

The study followed the STrengthening the Reporting of OBservational studies in Epidemiology (STROBE) reporting guideline.

All statistical analyses were performed using SAS (SAS Institute, Cary, NC, USA), R (R Foundation for Statistical Computing, Vienna, Austria), and Excel (Microsoft Corp., Redmond, WA, USA) software tools.

## RESULTS

A total of 764 respondents with tinnitus participated in the survey. Most participants came from Italy (*n* = 181), the United Kingdom (*n* = 181), the Netherlands (*n* = 152), Germany (*n* = 96), and Spain (*n* = 69). Our analysis is exclusively based on the 679 participants (89%) from these five countries, which had at least 20 individuals involved in the questionnaire each. Within this sample, the mean age was 57 (standard deviation [SD], 12.5) years; 365 (57.4%) were males and 271 (42.6%) were females; 122 (18.0%) reported slight, 260 (38.3%) moderate, and 297 (43.7%) severe tinnitus. Overall, 56.0% of participants were employed (including self-employment); 33.9% retired; and 10.2% either disabled, unable to work, or without employment. In terms of education, 11.2% had lower secondary, primary, or no formal education; 39.4% had an upper secondary level of education; and 46.9% held university education. Detailed breakdown of the sample according to selected participants’ characteristics by tinnitus severity is provided in Table 2.

**Table 2.** Descriptive statistics of 679 respondents from Italy, United Kingdom, Netherlands, Germany, and Spain, 2022

Characteristic	Tinnitus severity		

	Slight	Moderate	Severe
*N*	*122*	*260*	*297*

Age, years, mean (SD)	60.8 (13.3)	58.1 (11.9)	54.9 (12.3)

Age, years, %			
≤51	22.0	24.1	34.1
52–61	22.9	32.4	32.6
≥62	55.1	43.6	33.3

Sex, %			
Male	63.6	57.3	54.9
Female	36.4	42.7	45.1

Country, %			
Italy	32.8	28.9	22.2
United Kingdom	19.7	28.9	27.6
Netherlands	31.2	21.2	19.9
Germany	4.9	13.9	18.2
Spain	11.5	7.3	12.1

Occupation, %			
Disabled, not able to work	5.7	2.3	6.7
Employed	36.1	43.5	48.8
Not employed	4.1	4.2	6.7
Retired	41.8	38.9	26.3
Self-employed	12.3	11.2	11.5

Approximate annual personal income, compared to the average in your country, %			
Below average	45.9	30.0	27.6
Average	36.9	46.9	38.0
Above average	13.9	16.2	24.9

Level of education			
No school	0.0	0.0	0.9
Primary school	0.9	0.9	0.9
Lower secondary	11.8	11.7	13.5
Upper secondary	43.6	42.3	34.8
University or higher	43.6	45.1	50.0

Impact of COVID-19 on the number of treatments/doctor visits related to tinnitus			
Had no impact	89.8	65.4	52.6
Slightly increased	0.0	0.8	1.8
Moderately increased	0.9	0.8	1.1
Highly increased	1.9	1.7	6.6
Slightly reduced	3.7	9.3	7.7
Moderately reduced	0.9	7.2	11.8
Highly reduced	2.8	14.8	18.4

COVID-19, coronavirus disease 2019; SD, standard deviation.

If the sum does not equal 100% it is due to missing values or “prefer not to mention” answers.

Table 3 outlines OOP expenses encompassing healthcare visits, treatments, drugs, hearing aids or supporting systems and activities for the entire sample. Average number of annual healthcare visits was 0.96, 2.92, and 6.60 for slight, moderate, and severe tinnitus, respectively. Mean annual OOP expenses per person with tinnitus for healthcare visits were 104€ (95% CI, 44–186 €) for slight tinnitus, 313€ (95% CI, 175–581€) for moderate tinnitus, and 639€ (95% CI, 420–870€) for severe tinnitus. For treatments, OOP mean expenses per person increased with severity, totaling 42€ (95% CI, 2–153€), 159€ (95% CI, 56–381€), and 233€ (95% CI, 143–803€), respectively. Expenses for drugs were 3€ (95% CI, 0–14€), 17€ (95% CI, 6–37€), and 112€ (95% CI, 43–237€), while expenses for hearing aids or supporting systems were 54€ (95% CI, 24–102€), 76€ (95% CI, 45–125€), and 105€ (95% CI, 63–174€) for slight, moderate, and severe tinnitus, respectively. Activity-related expenses were 95€ (95% CI, 8–236€), 85€ (95% CI, 33–164€), and 293€ (95% CI, 90–605€) for slight, moderate, and severe tinnitus, respectively.

**Table 3.** Annual out-of-pocket (OOP) expenses for healthcare visits, treatments, drugs, hearing aids, or supporting systems and activities due to tinnitus, Europe 2022

Items	Tinnitus severity		

	Slight	Moderate	Severe
*n*	*122*	*260*	*297*


***HEALTHCARE VISITS***			

General Practitioner			
Proportion, %^a^	4.9%	18.8%	36.0%
Average number of visits	0.07	0.50	1.22
**Annual OOP mean expenses per person with tinnitus (€)**	**2 €**	**14 €**	**34 €**
Ear nose and throat specialist			
Proportion, %^a^	9.0%	21.5%	42.8%
Average number of visits	0.16	0.42	1.18
**Annual OOP mean expenses per person with tinnitus (€)**	**13 €**	**33 €**	**93 €**
Audiologist			
Proportion, %^a^	13.9%	19.2%	35.0%
Average number of visits	0.26	0.37	0.86
**Annual OOP mean expenses per person with tinnitus (€)**	**37 €**	**52 €**	**122 €**
Psychiatrist			
Proportion, %^a^	0.0%	2.3%	6.4%
Average number of visits	0.00	0.08	0.49
**Annual OOP mean expenses per person with tinnitus (€)**	**0 €**	**9 €**	**50 €**
Psychologist			
Proportion, %^a^	1.6%	7.3%	15.5%
Average number of visits	0.07	0.40	1.17
**Annual OOP mean expenses per person with tinnitus (€)**	**9 €**	**50 €**	**148 €**
Chiropractor			
Proportion, %^a^	0.8%	2.7%	6.1%
Average number of visits	0.03	0.17	0.33
**Annual OOP mean expenses per person with tinnitus (€)**	**4 €**	**20 €**	**39 €**
Physiotherapist			
Proportion, %^a^	2.5%	6.2%	12.1%
Average number of visits	0.26	0.67	0.94
**Annual OOP mean expenses per person with tinnitus (€)**	**17 €**	**42 €**	**59 €**
Company doctor			
Proportion, %^a^	0.8%	1.2%	4.0%
Average number of visits	0.03	0.03	0.12
**Annual OOP mean expenses per person with tinnitus (€)**	**0 €**	**0 €**	**0 €**
Social worker			
Proportion, %^a^	0.0%	1.2%	2.4%
Average number of visits	0.00	0.10	0.10
**Annual OOP mean expenses per person with tinnitus (€)**	**0 €**	**32 €**	**31 €**
Other healthcare provider^b^			
Proportion, %^a^	1.6%	2.7%	5.7%
Average number of visits	0.07	0.18	0.18
**Annual OOP mean expenses per person with tinnitus (€)**	**23 €**	**61 €**	**63 €**

TOTAL HEALTHCARE VISITS			
Average number of visits	0.96	2.92	6.60
**Annual OOP mean expenses per person with tinnitus (€) and 95% confidence interval** ^e^	**104 € (44 €; 186 €)**	**313 € (175 €; 581 €)**	**639 € (420 €; 870 €)**

***TREATMENTS***			

Counselling			
Proportion, %^a^	1.6%	5.0%	11.8%
Average number of treatments	0.07	0.20	0.46
**Annual OOP mean expenses per person with tinnitus (€)**	**3 €**	**10 €**	**24 €**
Psychotherapy			
Proportion, %^a^	1.6%	5.4%	11.4%
Average number of treatments	0.24	0.48	1.08
**Annual OOP mean expenses per person with tinnitus (€)**	**38 €**	**78 €**	**172 €**
Sound stimulation			
Proportion, %^a^	0.8%	2.7%	6.7%
Average number of treatments	0.01	0.05	0.30
**Annual OOP mean expenses per person with tinnitus (€)**	**1 €**	**3 €**	**19 €**
rTMS			
Proportion, %^a^	0.0%	0.8%	0.0%
Average number of treatments	0.00	0.08	0.00
**Annual OOP mean expenses per person with tinnitus (€)**	**0 €**	**58 €**	**0 €**
Neurostimulation			
Proportion, %^a^	0.0%	0.8%	2.0%
Average number of treatments	0.00	0.08	0.13
**Annual OOP mean expenses per person with tinnitus (€)**	**0 €**	**10 €**	**16 €**
Other treatment^b^			
Proportion, %^a^	0.8%	0.4%	1.3%
Average number of treatments	0.01	0.01	0.05
**Annual OOP mean expenses per person with tinnitus (€)**	**0 €**	**0 €**	**2 €**

TOTAL TREATMENTS			
Average number of treatments	0.32	0.89	2.01
**Annual OOP mean expenses per person with tinnitus (€) and 95% confidence interval** ^e^	**42 € (2 €; 153 €)**	**159 € (56 €; 381 €)**	**233 € (143 €; 803 €)**

***DRUGS***			

Alprazolam			
Proportion, %^a^	0.0%	0.8%	4.7%
Average number of drugs	0.00	0.00	0.22
**Annual OOP mean expenses per person with tinnitus (€)**	**0 €**	**0 €**	**2 €**
Diazepam			
Proportion, %^a^	0.0%	0.0%	4.7%
Average number of drugs	0.00	0.00	0.27
**Annual OOP mean expenses per person with tinnitus (€)**	**0 €**	**0 €**	**3 €**
Betahistine			
Proportion, %^a^	0.0%	1.9%	2.0%
Average number of drugs	0.00	0.08	0.13
**Annual OOP mean expenses per person with tinnitus (€)**	**0 €**	**1 €**	**2 €**
Mirtazapine			
Proportion, %^a^	0.0%	0.0%	3.4%
Average number of drugs	0.00	0.00	0.23
**Annual OOP mean expenses per person with tinnitus (€)**	**0 €**	**0 €**	**1 €**
Other drugs^b^			
Proportion, %^a^	0.8%	6.5%	21.9%
Average number of drugs	0.08	0.39	2.50
**Annual OOP mean expenses per person with tinnitus (€)**	**3 €**	**16 €**	**104 €**

TOTAL DRUGS			
Average number of drugs	0.08	0.47	3.35
**Annual OOP mean expenses per person with tinnitus (€) and 95% confidence interval** ^e^	**3 € (0 €; 14 €)**	**17 € (6 €; 37 €)**	**112 € (43 €; 237 €)**

***HEARING AIDS OR SUPPORTING SYSTEMS*** *^c^*			

Hearing support for tinnitus only			
Proportion, %^a^	5.7%	9.6%	14.5%
Annual depreciation^d^	25%	25%	25%
**Annual OOP mean expenses per person with tinnitus (€)**	**11 €**	**18 €**	**27 €**

Hearing support for hearing loss and tinnitus			
Proportion, %^a^	7.4%	10.0%	13.5%
Annual depreciation^d^	25%	25%	25%
**Annual OOP mean expenses per person with tinnitus (€)**	**43 €**	**58 €**	**78 €**

TOTAL HEARING AIDS OR SUPPORTING SYSTEMS			
**Annual OOP mean expenses per person with tinnitus (€) and 95% confidence interval** ^e^	**54 € (24 €; 102 €)**	**76 € (45 €; 125 €)**	**105 € (63 €; 174 €)**

***ACTIVITIES***			

Acupuncture			
Proportion, %^a^	2.5%	4.2%	11.8%
Average number of activities	0.61	0.41	0.73
**Annual OOP mean expenses per person with tinnitus (€)**	**69 €**	**47 €**	**82 €**
Homeopathy			
Proportion, %^a^	0.8%	1.9%	4.0%
Average number of activities	0.10	0.10	0.17
**Annual OOP mean expenses per person with tinnitus (€)**	**26 €**	**26 €**	**46 €**
Osteopathy			
Proportion, %^a^	0.0%	0.8%	2.7%
Average number of activities	0.00	0.05	0.70
**Annual OOP mean expenses per person with tinnitus (€)**	**0 €**	**13 €**	**165 €**

TOTAL ACTIVITIES			
Average number of activities	0.70	0.56	1.59
**Annual OOP mean expenses per person with tinnitus (€) and 95% confidence interval** ^e^	**95 € (8 €; 236 €)**	**85 € (33 €; 164 €)**	**293 € (90 €; 605 €)**

OOP, out-of-pocket; rTMS, repetitive transcranial magnetic stimulation.

^a^Proportions indicate the percentages of people who indicated the use of each item at least once in the previous year.

^b^“Other healthcare provider” includes several providers, such as dentists, oral surgeons and orthodontists, as well as gnathologists, endocrinologists and others. “Other treatment” includes several options, such as the use of bite, wax or haptotherapy. “Other drugs” includes several options, such as amitriptyline, cortisone, sertraline.

^c^We summed the individual purchase costs with the maintenance costs in local currency (eg, battery cost, repair cost), as indicated by the respondents.

^d^The question regarding hearing support systems did not specify annual expenses but total costs per device, therefore we assumed an average 4-year lifespan, and a 25% annual depreciation was applied to respondents’ indicated costs.

^e^Total annual OOP mean expenses’ percentile 95% confidence intervals were created on 5000 bootstrap replications.

eTable 1, eTable 2, eTable 3, eTable 4, eTable 5, eTable 6, and eTable 7 show OOP expenses of all items by sex and countries. In addition, eTable 8 investigates the OOP mean expenses of all items stratified by socio-economic status.

Table 4 sums up the OOP annual expenses per person with tinnitus, overall, by sex and country. The analysis revealed that overall annual expenses were 368€ (95% CI, 78–690€), 728€ (95% CI, 316–1,288€), and 1,492€ (95% CI, 760–2,688€) for slight, moderate, and severe tinnitus, respectively, which meant a weighted average expenditure (computed based on the distribution in the general population of tinnitus severity-specific prevalence from the ETS study) of 565€ annually for a person with any tinnitus. This amount is divided as follows: 209€ (37.1%) for healthcare visits, 93€ (16.4%) for treatments, 16€ (2.8%) for drugs, 64€ (11.4%) for hearing supporting tools, and 183€ (32.3%) for acupuncture, homeopathy, and osteopathy. OOP annual expenses were 544€ for males and 435€ for females and there were major variations across countries: 366€ in Italy, 214€ in the United Kingdom, 904€ in the Netherlands, 870€ in Germany, and 701€ in Spain.

**Table 4.** Total annual OOP expenses for tinnitus management overall, stratified by sex and country, Europe, 2022

Type	Severity			Any tinnitus

	Slight	Moderate	Severe	
Overall (*n* = 679)	*122*	*260*	*297*	
(A) OOP annual expenses per person with tinnitus^a^	368 € (78 €; 690 €)	728 € (316 €; 1,288 €)	1,492 € (760 €; 2,688 €)	565 €
(B) Proportion of people with tinnitus (ETS survey)	8.56%	4.25%	1.07%	13.88%
(C) People with tinnitus in 5 countries^c^	19,354,352	9,617,815	2,419,839	31,392,006
OOP expenses in 5 countries = A * C (M€)	7,128 M€ (5,751 M€; 8,552 M€)	7,004 M€ (5,172 M€; 9,130 M€)	3,610 M€ (1,832 M€; 5,885 M€)	17,742 M€

Male (*n* = 365)	*75*	*138*	*152*	
(A1) OOP annual expenses per person with tinnitus^b^	217 € (62 €; 526 €)	915 € (264 €; 2,057 €)	2,169 € (638 €; 4,202 €)	544 €
(B1) Proportion of people with tinnitus, males (ETS survey)	9.00%	3.67%	0.97%	13.64%
(C1) People with tinnitus in 5 countries, males^c^	9,916,138	4,043,936	1,073,134	15,033,207
OOP expenses in 5 countries, males = A1 * C1 (M€)	2,151 M€ (1,590 M€; 2,767 M€)	3,702 M€ (2,261 M€; 5,521 M€)	2,327 M€ (8,214 M€; 4,645 M€)	8,180 M€

Female (*n* = 271)	*43*	*103*	*125*	
(A2) OOP annual expenses per person with tinnitus^b^	336 € (14 €; 951 €)	457 € (178 €; 981 €)	1,030 € (519 €; 2,292 €)	435 €
(B2) Proportion of people with tinnitus, females (ETS survey)	8.13%	4.80%	1.17%	14.10%
(C2) People with tinnitus in 5 countries, females^c^	9,429,542	5,572,435	1,355,576	16,357,553
OOP expenses in 5 countries, females = A2 * C2 (M€)	3,173 M€ (2,339 M€; 4,0689 M€)	2,545 M€ (1,652 M€; 3,508 M€)	1,397 M€ (513 M€; 2,620 M€)	7,114 M€

IT (*n* = 181)	*40*	*75*	*66*	
(A3) OOP annual expenses per person with tinnitus^b^	71 € (7 €; 168 €)	615 € (111 €; 1,643 €)	2,075 € (409 €; 3,990 €)	366 €
(B3) Proportion of people with tinnitus, IT (ETS survey)	8.39%	4.46%	0.80%	13.65%
(C3) People with tinnitus in IT^c^	4,198,109	2,231,653	400,296	6,830,058
OOP expenses in IT = A3 * C3 (M€)	298 M€ (239 M€; 363 M€)	1,372 M€ (1,008 M€; 1,831 M€)	831 M€ (373 M€; 1,502 M€)	2,500 M€

UK (*n* = 181)	*24*	*75*	*82*	
(A4) OOP annual expenses per person with tinnitus^b^	89 € (5 €; 283 €)	348 € (71 €; 700 €)	883 € (185 €; 1,388 €)	214 €
(B4) Proportion of people with tinnitus, UK (ETS survey)	9.82%	4.55%	0.91%	15.28%
(C4) People with tinnitus in UK^c^	5,215,611	2,416,602	483,320	8,115,533
OOP expenses in UK = A4 * C4 (M€)	467 M€ (376 M€; 555 M€)	840 M€ (608 M€; 1,065 M€)	427 M€ (200 M€; 740 M€)	1,734 M€

NL (*n* = 152)	*38*	*55*	*59*	
(A5) OOP annual expenses per person with tinnitus^b^	452 € (56 €; 1,466 €)	1,198 € (129 €; 3,931 €)	2,919 € (907, 5,965 €)	904 €
(B5) Proportion of people with tinnitus, the NL (ETS survey)	8.67%	4.73%	1.25%	14.65%
(C5) People with tinnitus in the NL^c^	1,230,547	671,337	177,415	2,079,298
OOP expenses in the NL = A5 * C5 (M€)	556 M€ (521 M€; 591 M€)	805 M€ (740 M€; 877 M€)	518 M€ (433 M€; 608 M€)	1,879 M€

DE (*n* = 96)	*6*	*36*	*54*	
(A6) OOP annual expenses per person with tinnitus^b^	641 € (0 €; 2,396 €)	954 € (121 €; 3,726 €)	1,969 € (249 €; 5,832 €)	870 €
(B6) Proportion of people with tinnitus, DE (ETS survey)	6.46%	4.45%	1.01%	11.93%
(C6) People with tinnitus in DE^c^	4,494,401	3,095,988	702,685	8,293,073
OOP expenses in DE = A6 * C6 (M€)	2,880 M€ (2,216 M€; 3,627 M€)	2,955 M€ (2,112 M€; 3,951 M€)	1,384 M€ (658 M€; 2,452 M€)	7,219 M€

ES (*n* = 69)	*14*	*19*	*36*	
(A7) OOP annual expenses per person with tinnitus^b^	408 € (4 €; 7,382 €)	974 € (25 €; 3,639 €)	2,085 € (122 €; 6,916 €)	701 €
(B7) Proportion of people with tinnitus, ES (ETS survey)	10.73%	3.06%	1.67%	15.47%
(C7) People with tinnitus in ES^c^	4,215,684	1,202,236	656,122	6,074,043
OOP expenses in ES = A7 * C7 (M€)	1,720 M€ (1,424 M€; 2,004 M€)	1,171 M€ (793 M€; 1,626 M€)	1,368 M€ (797 M€; 2,037 M€)	4,259 M€

DE, Germany; ES, Spain; ETS, European Tinnitus Survey^12^; IT, Italy; NL, Netherlands; M€, million euros; OOP, out of pocket; UK, United Kingdom.

^a^Resulted from the sums of the out-of-pocket mean expenses per person with tinnitus of all the items in Table 1.

^b^Resulted from the sums of the out-of-pocket mean expenses per person with tinnitus of all the items in eTable 3, eTable 4, eTable 5, eTable 6, eTable 7, eTable 8, and eTable 9.

^c^Based on the adult population estimates by United Nations World Population Prospects 2021.

This table shows the total OOP in the sample of *n* = 679 individuals, as well as divided by sex (*n* = 365 males, *n* = 271 females) and country (IT *n* = 181, UK *n* = 181, NL *n* = 152, DE *n* = 96, ES *n* = 69). For each of these *n*, the following were calculated: the expenses per person represented by A (from A to A7), the weighted proportion of people with tinnitus according to the ETS survey estimates (from B to B7), and what that proportion meant in terms of number of people (from C to C7). Each of these elements was identified by stratifying for severity. To find the total OOP expenses, a multiplication was performed for each set (thus, A * C, A1 * C1, A2 * C2, and so on). The column indicating any tinnitus is the weighted average of the severity categories.

Average number of sick leave days per year also exhibited a gradient, with 0.5 days of sick leave within the previous 12 months for slight tinnitus, 1.6 days for moderate tinnitus, and 4.7 days for severe tinnitus (Table 5). Additionally, participants were queried about the portion of their monthly income they were willing to allocate towards alleviating tinnitus loudness/annoyance. A clear gradient emerged: individuals with slight, moderate, and severe tinnitus expressed a willingness to invest 1.6, 4.3, and 7.0 times their monthly income, respectively, to achieve complete relief (Table 5). A similar pattern was observed to achieve 50% reduction in annoyance, although to a lower extent.

**Table 5.** Description of sick days leave and willingness to pay among 679 people with tinnitus, Europe 2022

	Tinnitus severity		

	Slight	Moderate	Severe
*n*	*122*	*260*	*297*
***Sick days leave***			
*Percentage of those having any paid job within the past 12 months (percentage)*	57%	58%	66%
*Percentage of those who had a paid job within the past 12 months who were in sick leave due to tinnitus [a] (percentage)*	2.9%	10.3%	30.7%
Average days of sick leave = a * 15^a^	0.5	1.6	4.7

***Willingness to pay***			
*What part of your monthly income are you willing to pay to cure your tinnitus loudness/annoyance completely?* (mean)	1.6	4.3	7.0
*What part of your monthly income are you willing to pay to reduce your tinnitus loudness/annoyance by 50%?* (mean)	0.8	2.6	4.8

^a^Assuming for all severity categories average sick leave length 15 days, which was the overall mean in the sample.

The present table displays questions regarding quality of life, sick days taken, and willingness to pay. For quality of life, the average responses are indicated, stratified by tinnitus severity. As for sick days, the percentage was calculated among those who had paid employment in the previous 12 months and had taken sick days due to tinnitus. To calculate the average number of days across severity categories, the sample mean (15 days) was multiplied by the previously found percentage. Lastly, concerning willingness to pay, the two reported elements are the average responses to the two questionnaire questions as they are presented.

## DISCUSSION

Extrapolating from our results, we estimate that in our countries of interest each tinnitus person spends on average over 500€ per year—and people with severe tinnitus spend almost 1,500€ per year. Parts of these costs are spent for services for which there is no scientific evidence of efficacy. Noteworthy, we observed that the severity of the condition emerges as a pivotal factor influencing the expenses incurred by tinnitus sufferers. Major differences have been observed across countries.

The important pattern between expenses and tinnitus severity could be explained because, in the absence of a cure, as severity increases, people with tinnitus may find themselves “trying everything” and expending significant personal, mental, and economic efforts. The observed pattern was consistent with findings from a German study and a Dutch study, which showed that the socioeconomic^11^ and healthcare costs^4^ substantially increase with increasing tinnitus severity. This is true not only from a patient-centred perspective: in fact, several healthcare providers as well had recognized the low success rate of therapies for tinnitus, despite a large variety of treatment options.^5^

Irrespective of sex and country, the highest expenditure consistently stemmed from visits to healthcare providers. In agreement with current literature, we observed an important increase in healthcare providers’ utilization and corresponding expenses with increasing tinnitus severity.^4^^,^^11^ In particular, individuals experiencing severe tinnitus tended to visit healthcare providers, such as general practitioners and ear, nose, and throat specialists, more frequently. This notwithstanding, the main OOP expenses for healthcare visits come from audiologists and psychologists. This is likely due to the higher cost for visits and because these visits are less frequently reimbursed by the national health service or insurance providers.

Regarding treatments, not surprisingly,^17^ psychotherapy alone accounted for more than two thirds of total expenses, whereas neurostimulation and repetitive transcranial magnetic stimulation (rTMS) were not frequently used (by less than 5% and less than 1% of our sample, respectively). Drugs are only rarely prescribed, so expenses due to these items account for less than 5% of the whole OOP expenses, in agreement with at least one previous study.^4^ This observation aligns also with the fact that the majority of our participants exhibit chronic tinnitus, while the literature has demonstrated that, for acute subjective tinnitus, pharmacological prescriptions are common.^5^ Hearing aids or supporting systems have been reported by only around 10% of subjects with tinnitus.

It emerges that around one-third of the total expenditure is spent on interventions that are not recommended in the EU-guidelines or by the National Institute for Health and Care Excellence,^18^^,^^19^ such as acupuncture, homeopathy, and osteopathy. The use of acupuncture in adult patients has failed to demonstrate a clear benefit for tinnitus patients.^20^^,^^21^ To our knowledge, only one study focused specifically on the use of homeopathy in tinnitus treatment 25 years ago, showing to be equally effective as placebo.^22^ In the case of osteopathy, which should be defined as an umbrella term encompassing pseudoscientific subcategories and evidence-based therapeutic approaches,^23^ to our knowledge no RCT exists on its efficacy. Reviews on the use of osteopathic care for other complaints rather than tinnitus found poor methodological quality of studies in the field.^24^

Our estimates did not take into account dietary supplements. In our data 15.8%, 33.0%, 40.3% reported to use dietary supplements for their tinnitus at least monthly among slight, moderate, and severe tinnitus respondents, respectively. Despite the lack of information regarding the cost of such supplements, we believe it is important to highlight this result, as there is no evidence for dietary supplementation in tinnitus. When focusing on Cochrane-based evidence, the two most well-known supplements in the management of tinnitus (zinc^25^ and ginkgo biloba)^26^ have shown no benefits. Instead, several side effects have been observed, including allergic reactions, headaches, and gastrointestinal issues. For such reasons, it would be advisable to inform people with tinnitus about the state of the art in terms of proven effective therapies, as well as training professionals to follow the international guidelines available. Unfortunately, such supplements are often recommended by healthcare professionals, who in doing so, do not empower their patients to make informed choices.

Besides direct monetary costs, a further financial loss is work absenteeism: among those being employed, people with slight or moderate tinnitus lose around 1 day per year, while people with severe tinnitus almost 1 working week. Our results are in line with the existing literature: a Swedish study has observed that sick-leave spells for tinnitus and hearing loss can be remarkably long, extending in many cases for an entire year. Additionally, they tend to be more common among women.^27^

In our analyses, a decreased self-reported quality of life was associated with an increased tinnitus severity (eTable 9), in strong agreement with the literature.^28^ The assumption that with greater severity, the greater one’s financial commitment, is also supported by our results regarding willingness to pay, also known to increase with severity.^29^^–^^31^

Our results provide some interesting insights into sex differences. It emerges that females, although less prevalent in our sample, report more severe tinnitus. This finding is consistent with existing literature, which has already shown that gender greatly impacts the psychological burden, thus leading to greater severity.^32^ Calculations also revealed a significant geographical difference in terms of expenditure. While this can be explained in terms of the varying prevalence, it is true that the healthcare system model may to some extent contribute to lower direct patient spending in some countries that rely on a public system, compared to others based on compulsory insurance. Speculations in this sense might be made also in terms of gross domestic product per capita; future studies should check how care for tinnitus is provided and reimbursed in different countries.

We acknowledge certain limitations of this study; first, due to the limited number of respondents providing estimates for each item, we were obliged to make certain assumptions, a challenge that was amplified in stratified analysis by sex and country. Furthermore, our sample remains susceptible to sample bias, as patients were recruited from tinnitus specialists and patient associations. Particularly, it is conceivable that participants in our sample may be more predisposed than others to utilize healthcare services. However, within the context of providing a rough estimate of the total cost across the five countries under examination, this sampling method also enables us to approach a plausible estimation of the overall expenditure. In addition, because of its nature of being a cross-sectional study with self-reported information on medical expenses paid, recall bias should be mentioned as possible limitation of this study. Moreover, it was difficult, due to the complex nature of the calculations underlying the proposed estimates, to provide reliable measures of uncertainty to accompany the costs. We attempted to overcome this limitation performing bootstraps and reporting the confidence intervals. Furthermore, our calculations may substantially underestimate the accurate expenditure, as we decided to exclude certain items (eg, costs associated with sports, yoga, and similar activities). Additionally, we did not include the costs associated with any transportation means that may have been used to attend appointments or visits, because we acknowledged some difficulties in the interpretation of the questionnaire by participants, which resulted in unreliable responses. This approach allowed us to obtain conservative estimates. Finally, we do not have information on costs associated to causes of tinnitus, since this was out of the scope of the present analysis. We believe we were able to prevent respondents from indicating costs associated with anything other than tinnitus, explicitly asking in each question only to refer to expenses incurred for the management and treatment of the symptom of tinnitus and not for any other purpose.

Moreover, in our questionnaire we did not include questions regarding the cost of dietary supplements, and we neglected to inquire about expenses on hearing aids or supporting systems over the preceding 12 months. Consequently, it is evident that a refined and validated iteration of the questionnaire is imperative for future investigations. Ideally, this revised version should be structured to facilitate a comprehensive cost-of-illness analysis. We specifically requested OOP costs, recognizing that people typically lack insight into the true cost borne by the health system for a visit or treatment, and therefore we were only able to study the perspective of people with tinnitus. A notable strength of our study lies in being able to stratify OOP expenses across the three categories of tinnitus severity as defined by the ETS survey, as well as to interpolate national expenditure based on the previous prevalence data based on the same ETS questions. This substantially facilitates a nuanced assessment of OOP expenses. In fact, the distribution of severity specific tinnitus prevalence in the general population of each country, applied to the OOP costs of people with tinnitus, allowed us to obtain the OOP costs of a person with tinnitus in each country.

In summary, this study represents the first investigation providing data on the OOP expenses of people with tinnitus. Building upon a preceding study, we were able to approximate the mean annual OOP expenditure for tinnitus in Italy, the United Kingdom, the Netherlands, Germany, and Spain, collectively surpassing 17 billion €. We believe that our efforts can contribute to the evidence that individuals with tinnitus are often compelled to spend a significant portion of their personal funds seeking relief for a symptom that lacks an effective cure. The scale of severe tinnitus expenses rivals that of severe forms of back pain^33^ and migraine,^34^ which are among the most prevalent disabilities worldwide.^2^^,^^35^ This translates to an exorbitant financial burden for people dealing with tinnitus.

## SUPPLEMENTARY MATERIAL

eMaterial 1. Survey on tinnitus out-of-pocket expenses

eTable 1. Out-of-pocket expenses for healthcare visits, treatments, drugs, hearing aids and activities, males only, Europe 2022

eTable 2. Out-of-pocket expenses for healthcare visits, treatments, drugs, hearing aids and activities, females only, Europe 2022

eTable 3. Out-of-pocket expenses for healthcare visits, treatments, drugs, hearing aids and activities, Italy only

eTable 4. Out-of-pocket expenses for healthcare visits, treatments, drugs, hearing aids and activities, United Kingdom only

eTable 5. Out-of-pocket expenses for healthcare visits, treatments, drugs, hearing aids and activities, Netherlands only

eTable 6. Out-of-pocket expenses for healthcare visits, treatments, drugs, hearing aids and activities, Germany only

eTable 7. Out-of-pocket expenses for healthcare visits, treatments, drugs, hearing aids and activities, Spain only

eTable 8. Out-of-pocket expenses for healthcare visits, treatments, drugs, hearing aids and activities, stratified by socioeconomic status, Europe 2022

eTable 9. Description of quality of life among 679 people with tinnitus, Europe 2022
