# Supplementary material for: The Out-of-pocket Expenses of People With Tinnitus in Europe
Source: J Epidemiol. 2024 Nov 5;34(11):515–25. doi: 10.2188/jea.JE20230358 (PMC11464849; doi:10.2188/jea.JE20230358)
Supplement: Supplementary file 1 [file je-34-515-s001.pdf]

eMaterial 1. Survey on tinnitus out-of-pocket expenses

Tinnitus socioeconomic cost study

Introduction:

Dear participant, with this survey we hope to gain insight into the financial burden to the individual, as well as to society, associated with suffering from tinnitus with the final aim to obtain an estimate of the health care costs associated with tinnitus.

Tinnitus is the repeated or constant perception of a sound in one or both ears (or head) which nobody else hears nor originates from a sound source in the environment. Oftentimes a ringing or buzzing sound is reported. From now this point onwards we will use the term tinnitus, which can mean any sound you hear repeatedly or constantly in your ears (in the head). We would like to kindly ask you to reply to the questions below as accurately as possible. Your answers will be stored safely and anonymously following the General Data Protection Regulation (GDPR) rules.

The approximate time to complete this questionnaire is estimated to 20 minutes.

Acknowledgements: UNITI project has received funding from the European Union's Horizon 2020 Research and Innovation Programme, Grant Agreement Number 848261

☐ I Agree to participate in the study and I Agree with the UNITI data privacy policy

1. **Have you participated in the UNITI clinical trials?**
   ☐ Yes (if yes, questions 2 to 6, 15 to 18 shall be skipped) **(Your answers below should refer to the past 12 months from the day you have been recruited to the UNITI RCTs)**

   Please enter your RCT identifier number: ………………….

   ☐ No

In Part A, we will ask you a few demographic questions. Please remember that our study is fully anonymized, and the data collection follows the GDPR principles.

Part A – Individual characteristics

2. **Age (in years)**: ......
3. **Sex:**
   ☐ Male

   ☐ Female

   ☐ Intersex

   ☐ I prefer not to say

4. **What is your height? ....** cm
5. **What is your weight? ....** kg
6. **Level of education:**
   ☐ No school

   ☐ Primary (elementary school)

☐ Lower secondary (middle school)

☐ Upper secondary (high school)

☐ University or higher degree

7. **Approximate annual personal income, compared to the average in your country:**
   ☐ Below average

   ☐ Average

   ☐ Above average

   ☐ Prefer not to mention

8. **Country of residence:** …………….
9. **Occupation**:
   ☐ Employed

   ☐ Self-Employed

   ☐ Not employed

   ☐ Retired

   ☐ Disabled, not able to work

10. **Are/were you exposed to occupational noise*?**

Occupational noise is the amount of acoustic energy received by an employee's auditory system when working in industry. An example can be the exposure to loud sounds/high noise levels

☐ Yes, if yes, please indicate the average number of working days per week…..

☐ No

☐ Not sure

Thank you for completing Part A. Part B consists of general questions about your overall hearing condition. In particular, we will ask you a few questions on hearing issues and on the sensation of noise in head or in one or both ears, which is a symptom that medical doctors call "tinnitus".

Part B – Overall hearing condition

11. **Over the past year, have you had noises (such as ringing or buzzing) in your head or in one or both ears that last for more than five minutes at a time?**
    ☐ Yes, most or all of the time

    ☐ Yes, a lot of the time

    ☐ Yes, some of the time

    ☐ No, not in the past year

    ☐ No, never

☐ Do not know/Prefer not to answer

12. **Over the past year, how much did these noises in your head or ears worry, annoy or upset you when they were at their worst?**
☐ Severely

☐ Moderately

☐ Slightly

☐ Not at all

☐ Do not know/Prefer not to answer

13. **Over the past year, have you seen your family doctor or another healthcare professional for problems with noises in your head or ears?**
☐ Yes, 5 or more visits

☐ Yes, 2 to 4 visits

☐ Yes, one visit

☐ No

☐ Do not know/Prefer not to answer

14. **Do you currently have any other difficulty with your hearing, such as listening to speech in a noisy situation?**
☐ Yes, cannot hear at all

☐ Yes, severe difficulty

☐ Yes, moderate difficulty

☐ Yes, slight difficulty

☐ No difficulty

☐ Do not know/Prefer not to answer

Thank you for completing Part B. Now, in Part C, we will ask you a few questions on "tinnitus"

Part C: Tinnitus diagnosis and characteristics

15. **How long ago did your tinnitus appear?**
…..Months ….Years

☐ Don't know

16. **How often do you have tinnitus on average?**
☐ Daily or almost daily

☐ Almost weekly

☐ Almost monthly

☐ Every few months

☐ Yearly

17. What best describes your tinnitus during a day?

☐ Constant: you can always or usually hear it in a quiet room

☐ Intermittent: "comes and goes", cannot always hear it in a quiet room

18. Where do you perceive your tinnitus?

☐ Right ear

☐ Left ear

☐ Both ears, worse in right

☐ Both ears, worse in left

☐ Both ears, equally

☐ Inside the head

☐ Other. Please specify (multiple answers are possible using comma)

____

☐ Do not know

19. Please indicate what bothers you the most about the tinnitus perception, multiple answers are possible.

☐ Sense of loss of control over my body/feeling like I'm sick

☐ Intrusive tinnitus

☐ Inability to concentrate

☐ Bad sleep quality

☐ Inability to ignore tinnitus

☐ Other, please specify (multiple answers are possible using comma)

……………………………………

20. Has a health care provider ever evaluated/diagnosed your tinnitus?

☐ Yes

☐ No

21. When was the first time a health care provider evaluated/diagnosed your tinnitus?

…..Month ….Year

☐ N/A

22. Please indicate which health care provider evaluated/diagnosed your tinnitus

☐ General practitioner

☐ ENT

☐ Psychiatrist

☐ Psychologist

☐ Audiologist

☐ Other, please specify……

23. **How severe were your tinnitus complaints when you noticed it in the beginning?**

☐ No distress, no impairment

☐ Tinnitus impairs (e. g. emotion, cognition, attention, task performance) occasionally/ occurs under stressful situations and mainly in silence

☐ Tinnitus regularly impairs (e. g. emotion, cognition, attention, task performance) occurs in several situations

☐ Tinnitus constantly leads to impairment (e. g. in emotion, cognition, attention, task and daily life interference) occurs in all situations

24. **How severe would you rate your tinnitus now?**

☐ No distress, no impairment

☐ Tinnitus impairs (e. g. emotion, cognition, attention, task performance) occasionally/ occurs under stressful situations and mainly in silence

☐ Tinnitus regularly impairs (e. g. emotion, cognition, attention, task performance) occurs in several situations

☐ Tinnitus constantly leads to impairment (e. g. in emotion, cognition, attention, task and daily life interference) occurs in all situations

25. **Do you suffer from any of the following illnesses or chronic physical or psychological complaints?**

☐ None

☐ Fibromyalgia

☐ Chronic low/back pain

☐ Irritable bowel syndrome

☐ Inflammatory rheumatic disorder

☐ Depression

☐ Migraines

☐ Allergies

☐ Asthma

☐ Headache

☐ Temporomandibular joint dysfunction (TMJ) disorder

☐ Vertigo

☐ Insomnia

☐ Hyperacusis

☐ Neck pain

☐ Ear pain

☐ Gastro-intestinal problems

☐ Sexual disfunction

☐ Other, please specify (multiple answers are possible using comma) ……

26. **How many first degree relatives do you have and how many of those do you know to have tinnitus and/or hearing loss?**
First degree relatives

..... Brother(s) ..... Sister(s)

...... Son(s) ..... Daughter(s)

First degree relatives with tinnitus or hearing loss

..... Father ……Mother

..... Brother(s) ..... Sister(s)

...... Son(s) ..... Daughter(s)

Thank you for completing Part C. Now, in Part D we will ask you a few questions about any treatment you followed due to tinnitus and the respective costs. Here, we would like you to provide us with your most accurate estimations.

Part D: Direct medical and non-medical costs

27. **When was the first and last time you received treatment because of tinnitus?** ***You can indicate here the period you visited a health care provider (i.e. General practitioner, ENT, Psychiatrist, Psychologist, Chiropractor, Physiotherapist, Social worker, Company doctor, Audiologist) for your treatment or the first time you used a hearing aid or had other form of treatment (i.e. use of drugs such as: Nitrazepam, Temazepam, Oxazepam, Lormetazepam, Zolpidem, Alprazolam etc, Magnetic stimulation, Neurostimulation, Repetitive Transcranial Magnetic Stimulation (rTMS or magnet therapy), Psychotherapy, Counselling, Sound stimulation)?***

First time: …..Month ….Year ☐ NA

Last time: …..Month ….Year ☐ NA

28. **Do you still experience tinnitus?**

☐ Yes, more severely than the last time I received treatment

☐ Yes, less severely than the last time I received treatment

☐ Yes, with the same severity than the last time I received treatment

☐ No

29. **If you received any <u>treatment because of tinnitus</u> within the past 12 months, how many appointments with a health care provider (i.e. General practitioner, ENT, Psychiatrist, Psychologist, Chiropractor, Physiotherapist, Social worker, Company doctor, Audiologist) for your treatment you had and/or other form of treatments (i.e Magnetic stimulation, Neurostimulation, Repetitive Transcranial Magnetic Stimulation-rTMS or magnet therapy-, Psychotherapy, Counselling, Sound stimulation, etc)?**

…….. number of appointments

30. **For how many hours per week did you have an appointment <u>relevant to your treatment</u> within the past 12 months? (if you have had multiple appointments, enter an average otherwise enter 0)**

__________ hours per week

31. **Have you been in contact with any of the following a health care provider <u>because of tinnitus</u> in the past 12 months? If yes, please indicate [a] the number of appointments in the past 12 months, [b] the average individual cost contribution per appointment, [c] the means of transport you usually used for the visit(s) and [d] the number of times you were accompanied by one or more family member(s). Multiple answers are possible.**

	**Yes/No**	**Indicate the number of appointment (in the past 12 months)**	**Average individual cost contribution per appointment (out-of-pocket costs) in local currency (You have to report the costs that were not covered by the National Health care System or by private insurance)**	**Means of transport (car, bicycle, on foot, public transport, other) you usually used for the visit(s)**	**Were you accompanied by one or more family member(s)? If yes, indicate how many times (in numbers)**
General practitioner					
ENT					
Psychiatrist					
Psychologist					
Chiropractor					
Physiotherapist					
Social worker					
Company doctor					
Audiologist (in the hospital or in an					

audiology center)					
Other healthcare provider, please specify specialism:<br>1….<br>2…					

32. **Did you receive any treatments <u>because of tinnitus</u> in the past 12 months? Multiple answers are possible**

	**Yes/No**	**Indicate the number of appointments (in the past 12 months)**	**Average individual cost contribution per appointment (out-of-pocket costs) in local currency (You have to report the costs that were not covered by the National Health care System or by private insurance)**	**Means of transport (car, bicycle, on foot, public transport, other) you usually used for the visit(s)**
Magnetic stimulation				
Neurostimulation				
Repetitive Transcranial Magnetic Stimulation (rTMS or magnet therapy)				
Psychotherapy				
Counselling				
Sound stimulation				
Other treatment, please specify:<br>1….<br>2….				

33. **Have you undertaken other activities <u>to reduce tinnitus</u> in the past 12 months? Multiple answers are possible**

	**Yes/No**	**Indicate the frequency (number of times) in the past 12 months**	**Average individual cost contribution in local currency (You have to report the costs that were not covered by the National Health care System or by private insurance) (Please specify if individualcosts are calculated per month or per session)**	**Means of transport (car, bicycle, on foot, public transport, other) you usually used for the visit(s)**
Sports center / sports club membership				
Yoga / relaxation therapy				
Homeopathy				
Acupuncture				
Running				

Hiking				
Other, please specify:<br>1….<br>2….				

33.1 Do you follow any special diet to reduce tinnitus?

☐ Yes

☐ No

33.2 Do you use any supplements (i.e. vitamins A, B complex, D, E, K, zinc, magnesium, iron) to reduce tinnitus?

☐ Yes, daily

☐ Yes, Weekly

☐ Yes, monthly

☐ No

34. Have you used prescription drugs in the past 12 months <u>because of tinnitus</u>?

☐ No

☐ Yes, if yes, which prescription drugs did you use, how many packages did you purchase the past year and how much was your individual contribution (personal costs not covered by the National Health care System/Private Insurance) per package in local currency (multiple answers possible), if it is fully covered by National Health care System or private insurance please indicate 0 in the individual contribution per package:

☐ **Nitrazepam** (e.g. Mogadon)

......number of packages purchased

….. individual contribution per package

☐ **Temazepam** (e.g. Normison)

......number of packages purchased

….. individual contribution per package

☐ **Oxazepam** (e.g. Seresta)

......number of packages purchased

….. individual contribution per package

☐ **Lormetazepam** (e.g Loramet)

......number of packages purchased

….. individual contribution per package

☐ **Zolpidem** (e.g Stilnoct)

......number of packages purchased

….. individual contribution per package

☐ **Alprazolam** (e.g. Xanax)

......number of packages purchased

….. individual contribution per package

☐ **Lorazepam** (e.g. Temesta)

......number of packages purchased

….. individual contribution per package

☐ **Diazepam** (e.g. Valium)

......number of packages purchased

….. individual contribution per package

☐ Other, namely ____

......number of packages purchased

….. individual contribution per package

35. Have you ever purchased a hearing aid (masker/noise generator - a hearing aid looking device, which makes a noise) <u>due to tinnitus and/or hearing loss</u>?

☐ No

☐ Yes, due to tinnitus, name the hearing aid ________

- Approximate individual purchase costs in local currency (not covered by the National Health care System/Private Insurance, if fully covered please indicate 0) ________
- Approximate maintenance costs in local currency (e.g. battery cost, repair cost, etc)________

☐ Yes, due to hearing loss, name the hearing aid ________

- Approximate individual purchase costs in local currency (not covered by the National Health care System/Private Insurance, if fully covered please indicate 0) ________
- Approximate maintenance costs in local currency (e.g. battery cost, repair cost, etc)________

☐ Yes, due to tinnitus and hearing loss, name the hearing aid ________

- Approximate individual purchase costs in local currency (not covered by the National Health care System/Private Insurance, if fully covered please indicate 0) ________
- Approximate maintenance costs in local currency (e.g. battery cost, repair cost, etc)________

36. **If you have used other drugs or aids in the past 12 months, or have performed other activities specifically <u>because of your tinnitus symptoms</u> or to ameliorate your tinnitus condition, you can describe them below. If there were costs involved, please write them down.**

**Activity / (tool) resource**	**Average individual contribution (not covered by the National Health care System/Private Insurance, if fully covered please indicate 0) in local currency**	**Possible maintenance costs in local currency**

Thank you for completing Part D. Now, in Part E we will ask you few questions regarding the part of your monthly income you are willing to pay to cure your tinnitus loudness/annoyance.

Part E: WTP

37. **What part of your monthly income are you willing to pay to cure your tinnitus loudness/annoyance completely?**
☐ 1/4 of my monthly income
☐ 1/2 of my monthly income
☐ Once my monthly income
☐ 2 times my monthly income
☐ 5 times my monthly income
☐ 10 times my monthly income
☐ 20 times my monthly income
☐ Over 20 times my monthly income

38. **What part of your monthly income are you willing to pay to reduce your tinnitus loudness/annoyance by 50%?**
☐ 1/4 of my monthly income
☐ 1/2 of my monthly income
☐ Once my monthly income
☐ 2 times my monthly income
☐ 5 times my monthly income
☐ 10 times my monthly income
☐ 20 times my monthly income

☐ Over 20 times my monthly income

39. Do you have a private insurance?

☐ Yes

☐ No

40. To help people describe how good or bad their state of health is we have drawn a scale similar to a thermometer on which 100 marks the best state of health imagined and 0 the worst state of health imagined. We would like you to indicate on this scale, in your opinion, how good or bad your state of health is today

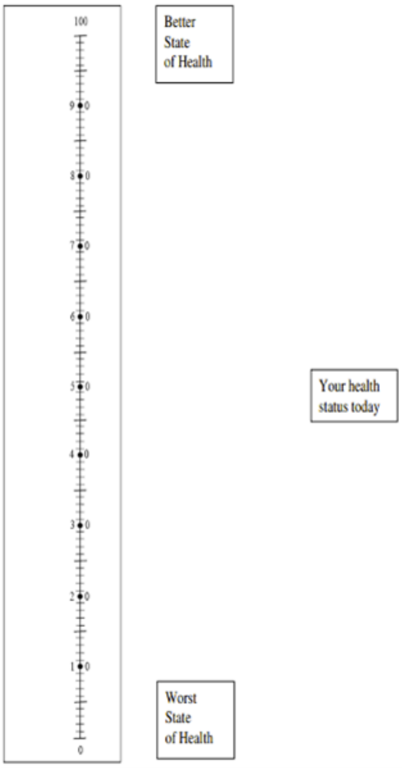

Thank you for completing part E. Now, in Part F we will ask you few questions regarding the effect of tinnitus into your working life. We would like you to provide us with your most accurate estimations.

Part F: Indirect costs

41. Did you have any paid job within the past 12 months?

☐ Yes

☐ No – go to question 45

42. Were you in sick leave <u>due to tinnitus</u> during the past 12 months?

☐ Yes

☐ No

43. How often were you on a sick leave <u>due to tinnitus</u> the past 12 months?

__________ times

44. **Can you indicate the approximate duration of these absence periods in the past 12 months <u>due to tinnitus</u>?**

Period 1: ____________ days / months)

Period 2:____________ days / months

Period 3:____________ days / months

Period 4:____________ days / months

45. **During a normal working day and while you experience tinnitus can you indicate on average how much your concentration level is affected?**

1	2	3	4	5	6	7	8	9	10
(Not at all)									(Completely)

46. **During a normal working day and while you experience tinnitus can you indicate on average how much your productivity level is affected?**

1	2	3	4	5	6	7	8	9	10
(Not at all)									(Completely)

Thank you for completing Part F. Our last part (part G) is focused on the impact of COVID-19 on your tinnitus-related treatment/doctor visits.

Part G: COVID-19

47. **Overall, the COVID-19 pandemic and the restrictive measures put in place to reduce the virus spread:**

☐ <u>Reduced</u> the number of treatments/doctor visits **<u>related to tinnitus.</u>**

☐ Slightly

☐ Moderately

☐ Highly

☐ <u>Increased</u> the number of treatments/doctor visits **<u>related to tinnitus.</u>**

☐ Slightly

☐ Moderately

☐ Highly

☐ Had no impact on the number of treatments/doctor visits **<u>related to tinnitus</u>**

Part H: Participation to future follow-up study

48. **If you are willing and available to participate in a follow-up survey for this study in the future, we would be very grateful. Please write your e-mail address here, if you allow us to contact you again**:...

eTable 1. Out-of-pocket expenses for healthcare visits, treatments, drugs, hearing aids and activities, males only, Europe 2022

Item	Tinnitus severity		
	Slight	Moderate	Severe
n	*75*	*138*	*152*
TOTAL HEALTHCARE VISITS			
Average number of visits	0.86	3.00	6.24
**Annual OOP mean expenses per person with tinnitus (€)**	**136 €**	**441 €**	**700 €**
TOTAL TREATMENTS			
Average number of treatments	0.09	0.98	2.29
**Annual OOP mean expenses per person with tinnitus (€)**	**25 €**	**294 €**	**563 €**
TOTAL DRUGS			
Average number of drugs	0.13	0.34	4.03
**Annual OOP mean expenses per person with tinnitus (€)**	**5 €**	**11 €**	**140 €**
TOTAL HEARING AIDS OR SUPPORTING SYSTEMS[a, b]			
**Annual OOP mean expenses per person with tinnitus (€)**	**51 €**	**69 €**	**70 €**
TOTAL ACTIVITIES			
Average number of activities	0.00	0.26	2.00
**Annual OOP mean expenses per person with tinnitus (€)**	**0 €**	**100 €**	**696 €**

OOP, out-of-pocket.

[a] We summed the individual purchase costs with the maintenance costs in local currency (eg, battery cost, repair cost), as indicated by the respondents.

[b] The question regarding hearing aids or support systems did not specify annual expenses but total costs per device, therefore we assumed an average 4-year lifespan, and a 25% annual depreciation was applied to respondents' indicated costs.

eTable 2. Out-of-pocket expenses for healthcare visits, treatments, drugs, hearing aids and activities, females only, Europe 2022

Item	Tinnitus severity		
	Slight	Moderate	Severe
n	*43*	*103*	*125*
TOTAL HEALTHCARE VISITS			
Average number of visits	1.26	3.15	7.75
**Annual OOP mean expenses per person with tinnitus (€)**	**81 €**	**239 €**	**614 €**
TOTAL TREATMENTS			
Average number of treatments	0.74	0.75	1.72
**Annual OOP mean expenses per person with tinnitus (€)**	**49 €**	**71 €**	**229 €**
TOTAL DRUGS			
Average number of drugs	0.00	0.70	2.74
**Annual OOP mean expenses per person with tinnitus (€)**	**0 €**	**7 €**	**11 €**
TOTAL HEARING AIDS OR SUPPORTING SYSTEMS[ab]			
**Annual OOP mean expenses per person with tinnitus (€)**	**82 €**	**95 €**	**95 €**
TOTAL ACTIVITIES			
Average number of activities	1.73	0.75	1.20
**Annual OOP mean expenses per person with tinnitus (€)**	**124 €**	**45 €**	**82 €**

OOP, out-of-pocket.

[a] We summed the individual purchase costs with the maintenance costs in local currency (eg, battery cost, repair cost), as indicated by the respondents.

[b] The question regarding hearing aids or support systems did not specify annual expenses but total costs per device, therefore we assumed an average 4-year lifespan, and a 25% annual depreciation was applied to respondents' indicated costs.

eTable 3. Out-of-pocket expenses for healthcare visits, treatments, drugs, hearing aids and activities, Italy only

Item	Tinnitus severity		
	Slight	Moderate	Severe
n	*40*	*75*	*66*
TOTAL HEALTHCARE VISITS			
Average number of visits	0.35	2.05	6.66
**Annual OOP mean expenses per person with tinnitus (€)**	**39 €**	**205 €**	**660 €**
TOTAL TREATMENTS			
Average number of treatments	0.00	0.29	2.06
**Annual OOP mean expenses per person with tinnitus (€)**	**0 €**	**295 €**	**1,231 €**
TOTAL DRUGS			
Average number of drugs	0.00	0.45	2.03
**Annual OOP mean expenses per person with tinnitus (€)**	**0 €**	**12 €**	**60 €**
TOTAL HEARING AIDS OR SUPPORTING SYSTEMS[ab]			
**Annual OOP mean expenses per person with tinnitus (€)**	**32 €**	**82 €**	**106 €**
TOTAL ACTIVITIES			
Average number of activities	0.00	0.08	0.12
**Annual OOP mean expenses per person with tinnitus (€)**	**0 €**	**10 €**	**15 €**

OOP, out-of-pocket.

[a] We summed the individual purchase costs with the maintenance costs in local currency (eg, battery cost, repair cost), as indicated by the respondents.

[b] The question regarding hearing aids or support systems did not specify annual expenses but total costs per device, therefore we assumed an average 4-year lifespan, and a 25% annual depreciation was applied to respondents' indicated costs.

eTable 4. Out-of-pocket expenses for healthcare visits, treatments, drugs, hearing aids and activities, United Kingdom only

Item	Tinnitus severity		
	Slight	Moderate	Severe
n	*24*	*75*	*82*
TOTAL HEALTHCARE VISITS			
Average number of visits	0.63	1.67	4.42
**Annual OOP mean expenses per person with tinnitus (€)**	**32 €**	**99 €**	**242 €**
TOTAL TREATMENTS			
Average number of treatments	0.21	0.32	1.66
**Annual OOP mean expenses per person with tinnitus (€)**	**7 €**	**10 €**	**47 €**
TOTAL DRUGS			
Average number of drugs	0.00	0.32	2.61
**Annual OOP mean expenses per person with tinnitus (€)**	**0 €**	**4 €**	**25 €**
TOTAL HEARING AIDS OR SUPPORTING SYSTEMS[ab]			
**Annual OOP mean expenses per person with tinnitus (€)**	**50 €**	**139 €**	**156 €**
TOTAL ACTIVITIES			
Average number of activities	0.00	0.14	0.29
**Annual OOP mean expenses per person with tinnitus (€)**	**0 €**	**15 €**	**33 €**

OOP, out-of-pocket. [a] We summed the individual purchase costs with the maintenance costs in local currency (eg, battery cost, repair cost), as indicated by the respondents.

[b] The question regarding hearing aids or support systems did not specify annual expenses but total costs per device, therefore we assumed an average 4-year lifespan, and a 25% annual depreciation was applied to respondents' indicated costs.

eTable 5. Out-of-pocket expenses for healthcare visits, treatments, drugs, hearing aids and activities, Netherlands only

Item	Tinnitus severity		
	Slight	Moderate	Severe
n	*38*	*55*	*59*
TOTAL HEALTHCARE VISITS			
Average number of visits	0.98	2.35	8.27
**Annual OOP mean expenses per person with tinnitus (€)**	**180 €**	**510 €**	**1,385 €**
TOTAL TREATMENTS			
Average number of treatments	0.16	1.14	2.67
**Annual OOP mean expenses per person with tinnitus (€)**	**82 €**	**499 €**	**1,126 €**
TOTAL DRUGS			
Average number of drugs	0.26	0.76	3.59
**Annual OOP mean expenses per person with tinnitus (€)**	**13 €**	**40 €**	**177 €**
TOTAL HEARING AIDS OR SUPPORTING SYSTEMS[ab]			
**Annual OOP mean expenses per person with tinnitus (€)**	**58 €**	**65 €**	**118 €**
TOTAL ACTIVITIES			
Average number of activities	0.18	0.08	0.38
**Annual OOP mean expenses per person with tinnitus (€)**	**26 €**	**12 €**	**35 €**

OOP, out-of-pocket.

[a] We summed the individual purchase costs with the maintenance costs in local currency (eg, battery cost, repair cost), as indicated by the respondents.

[b] The question regarding hearing aids or support systems did not specify annual expenses but total costs per device, therefore we assumed an average 4-year lifespan, and a 25% annual depreciation was applied to respondents' indicated costs.

eTable 6. Out-of-pocket expenses for healthcare visits, treatments, drugs, hearing aids and activities, Germany only

Item	Tinnitus severity		
	Slight	Moderate	Severe
n	*6*	*36*	*54*
HEALTHCARE VISITS			
TOTAL HEALTHCARE VISITS			
Average number of visits	6.17	7.67	8.16
**Annual OOP mean expenses per person with tinnitus (€)**	**284 €**	**658 €**	**693 €**
TOTAL TREATMENTS			
Average number of treatments	4.67	1.67	1.64
**Annual OOP mean expenses per person with tinnitus (€)**	**194 €**	**65 €**	**53 €**
TOTAL DRUGS			
Average number of drugs	0.00	0.17	0.81
**Annual OOP mean expenses per person with tinnitus (€)**	**0 €**	**21 €**	**65 €**
TOTAL HEARING AIDS OR SUPPORTING SYSTEMS[ab]			
**Annual OOP mean expenses per person with tinnitus (€)**	**163 €**	**45 €**	**82 €**
TOTAL ACTIVITIES			
Average number of activities	0.00	0.55	2.95
**Annual OOP mean expenses per person with tinnitus (€)**	**0 €**	**161 €**	**1,074 €**

OOP, out-of-pocket.

[a] We summed the individual purchase costs with the maintenance costs in local currency (eg, battery cost, repair cost), as indicated by the respondents.

[b] The question regarding hearing aids or support systems did not specify annual expenses but total costs per device, therefore we assumed an average 4-year lifespan, and a 25% annual depreciation was applied to respondents' indicated costs.

eTable 7. Out-of-pocket expenses for healthcare visits, treatments, drugs, hearing aids and activities, Spain only

Item	Tinnitus severity		
	Slight	Moderate	Severe
n	*14*	*19*	*36*
TOTAL HEALTHCARE VISITS			
Average number of visits	1.14	3.41	6.93
**Annual OOP mean expenses per person with tinnitus (€)**	**98 €**	**576 €**	**810 €**
TOTAL TREATMENTS			
Average number of treatments	0.00	2.79	2.71
**Annual OOP mean expenses per person with tinnitus (€)**	**0 €**	**338 €**	**1,077 €**
TOTAL DRUGS			
Average number of drugs	0.00	0.74	7.57
**Annual OOP mean expenses per person with tinnitus (€)**	**0 €**	**11 €**	**167 €**
TOTAL HEARING AIDS OR SUPPORTING SYSTEMS[ab]			
**Annual OOP mean expenses per person with tinnitus (€)**	**0 €**	**34 €**	**19 €**
TOTAL ACTIVITIES			
Average number of activities	0.52	0.04	0.08
**Annual OOP mean expenses per person with tinnitus (€)**	**310 €**	**15 €**	**12 €**

OOP, out-of-pocket.

[a] We summed the individual purchase costs with the maintenance costs in local currency (eg, battery cost, repair cost), as indicated by the respondents.

[b] The question regarding hearing aids or support systems did not specify annual expenses but total costs per device, therefore we assumed an average 4-year lifespan, and a 25% annual depreciation was applied to respondents' indicated costs.

eTable 8. Out-of-pocket expenses for healthcare visits, treatments, drugs, hearing aids and activities, stratified by socioeconomic status, Europe 2022

Item	Socioeconomic status		
	Below average	Average	Above average
n	*133*	*280*	*216*
TOTAL HEALTHCARE VISITS			
Average number of visits	3.96	4.04	4.62
**Annual OOP mean expenses per person with tinnitus (€)**	**388 €**	**405 €**	**499 €**
TOTAL TREATMENTS			
Average number of treatments	1.35	1.26	1.37
**Annual OOP mean expenses per person with tinnitus (€)**	**293 €**	**272 €**	**239 €**
TOTAL DRUGS			
Average number of drugs	1.53	1.32	2.01
**Annual OOP mean expenses per person with tinnitus (€)**	**38 €**	**44 €**	**83 €**
TOTAL HEARING AIDS OR SUPPORTING SYSTEMS[ab]			
**Annual OOP mean expenses per person with tinnitus (€)**	**83 €**	**83 €**	**97 €**
TOTAL ACTIVITIES			
Average number of activities	0.84	1.38	0.89
**Annual OOP mean expenses per person with tinnitus (€)**	**117 €**	**223 €**	**157 €**

OOP, out-of-pocket.

[a] We summed the individual purchase costs with the maintenance costs in local currency (eg, battery cost, repair cost), as indicated by the respondents.

[b] The question regarding hearing aids or support systems did not specify annual expenses but total costs per device, therefore we assumed an average 4-year lifespan, and a 25% annual depreciation was applied to respondents' indicated costs. The sum is not equal 679 individuals, as not all respondents mentioned their socio-economic status.

eTable 9. Description of quality of life among 679 people with tinnitus, Europe 2022

	Tinnitus severity		
	Slight	Moderate	Severe
n	*122*	*260*	*297*
Quality of life			
If 100 marks the best state of health imagined and 0 the worst state of health imagined, indicate on this scale, in your opinion, how good or bad your state of health is today (mean)	72.7	64.8	53.5
